# Genome-Wide Transcriptional Profiles of the Berry Skin of Two Red Grape Cultivars (*Vitis vinifera*) in Which Anthocyanin Synthesis Is Sunlight-Dependent or -Independent

**DOI:** 10.1371/journal.pone.0105959

**Published:** 2014-08-26

**Authors:** Ben-Hong Wu, Yue-Gang Cao, Le Guan, Hai-Ping Xin, Ji-Hu Li, Shao-Hua Li

**Affiliations:** 1 Beijing Key Laboratory of Grape Science and Enology, and CAS Key Laboratory of Plant Resources, Institute of Botany, The Chinese Academy of Sciences, Beijing, P. R. China; 2 University of Chinese Academy of Sciences, Beijing, P. R. China; 3 Key Laboratory of Plant Germplasm Enhancement and Speciality Agriculture, Wuhan Botanical Garden, The Chinese Academy of Sciences, Wuhan, P. R. China; Key Laboratory of Horticultural Plant Biology (MOE), China

## Abstract

Global gene expression was analyzed in the berry skin of two red grape cultivars, which can (‘Jingyan’) or cannot (‘Jingxiu’) synthesize anthocyanins after sunlight exclusion from fruit set until maturity. Gene transcripts responding to sunlight exclusion in ‘Jingyan’ were less complex than in ‘Jingxiu’; 528 genes were induced and 383 repressed in the former, whereas 2655 genes were induced and 205 suppressed in ‘Jingxiu’. They were regulated either in the same or opposing manner in the two cultivars, or in only one cultivar. In addition to *VvUFGT* and *VvMYBA1*, some candidate genes (e.g. *AOMT, GST*, and *ANP*) were identified which are probably involved in the differential responses of ‘Jingxiu’ and ‘Jingyan’ to sunlight exclusion. In addition, 26 *MYB*, 14 *bHLH* and 23 *WD40* genes responded differently to sunlight exclusion in the two cultivars. Interestingly, all of the 189 genes classified as being relevant to ubiquitin-dependent protein degradation were down-regulated by sunlight exclusion in ‘Jingxiu’, but the majority (162) remained unchanged in ‘Jingyan’ berry skin. It would be of interest to determine the precise role of the ubiquitin pathway following sunlight exclusion, particularly the role of *COP9* signalosome, cullins, *RING-Box* 1, and *COP1*-interacting proteins. Only a few genes in the light signal system were found to be regulated by sunlight exclusion in either or both cultivars. This study provides a valuable overview of the transcriptome changes and gives insight into the genetic background that may be responsible for sunlight-dependent versus -independent anthocyanin biosynthesis in berry skin.

## Introduction

Anthocyanins, which are derived from the phenylpropanoid pathway, are a class of secondary metabolites that contribute to the red, blue, and purple coloring of a diverse range of flowers and the skin and flesh of fruit, as well as leaves, shoots, roots, and seeds [1]. Among other environmental factors, light is a critical stimulus regulating anthocyanin accumulation and the effect of light and shade on anthocyanin accumulation has been widely studied [1]–[4]. In general, anthocyanin accumulation is reduced under low light conditions and increased under high light in the fruit of many crops, including grapes [5]–[13], although too much radiation in the ultraviolet-B (UV-B) wavelength range can inhibit anthocyanin synthesis [14].

Anthocyanin production requires a number of genes, the most studied of which are the structural genes encoding the biosynthetic enzymes and the *R2R3 MYB* regulator family. In various tissues of *Arabidopsis*, *AtMYB11*, *AtMYB12* and *AtMYB111* together regulate the early anthocyanin biosynthetic genes chalcone synthase (*CHS*), chalcone isomerase (*CHI*) and flavanone 3-hydroxylase (*F3H*) in response to light [15]–[17]. In grapes, shade also suppresses and retards the accumulation of *CHS*, *CHI*, *F3H*, *DFR* (dihydroflavonol 4-reductase), *LDOX* (leucoanthocyanidin dioxygenase), *UFGT* (UDP-glucose: flavonoid 3-O-glucosyltransferase) and *VvMYBA1* mRNA [11]. *MYB* regulators often regulate these structural genes by activating their promoters. PcMYB can interact with light-regulatory unit 1 (*LRU1*), comprising an ACGT-containing element (ACE) and an *MYB* recognition element (MRE), which is necessary to mediate light-dependent activation of *CHS* in *Petroselinum crispum* [18]. PfMYBP1 was able to bind to the *DFR* gene promoter and its expression was induced by light in *Perilla frutescens* [19]. When fruit grown in the dark were exposed to sunlight, *MdMYB1* transcript levels increased over several days, correlating with anthocyanin synthesis in apple skin via the activation of the *MdDFR* and *MdUFGT* promoters [20]. Two other families of regulators, the basic helix-loop-helix (*bHLH*, also called *MYC*) and tryptophan-aspartic acid repeat (*WDR*), are involved in the function of the *MYB* family [21], [22], although they are less studied with respect to light-induced anthocyanin accumulation. They are not very sensitive to sunlight in *Petunia* [23] and the *MYC* gene is constitutively expressed in maize [24].

In addition, many studies on light-induced anthocyanins focus on their photoinduction, which involves three major classes of photoreceptors, namely phytochromes (PHY) for far-red and red, crytochromes (CRY)/phototropins (PHOT) for blue and ultraviolet-A (UV-A), and UV-B light receptors [25]–[28]. These photoreceptors often function by inducing the expression of anthocyanin biosynthesis genes [3]. The expression of some anthocyanin biosynthesis genes, specifically *CHS* and *F3H*, is induced by exposure to UV-A and is mediated by a distinct UV-A-specific photoreceptor [29]. *CHS* is also dependent on PHY-cGMP signaling [30]–[32]. PIF3 (phytochrome-interacting factor 3) and another transcription factor, HY5 (long hypocotyl 5), can positively regulate anthocyanin biosynthesis through direct binding to the promoters of the anthocyanin structural genes, including *CHS*, *CHI*, *F3H*, *F3’H* (flavonoid 3′-hydroxylase), *DFR* and *LDOX* [33]. Despite these insights, these studies often only focused on individual genes or small groups of genes related to anthocyanin biosynthesis. With respect to anthocyanin synthesis, a cDNA microarray analysis of green and colored berry skins of the grape ‘Shiraz’ showed a group of differentially expressed genes, including a diverse range of genes with unknown functions [34]. A genome-wide transcriptomic atlas of grapevine reveals that the growing organs, such as berry skin, were characterized by the high-level expression of a group of genes, including *F3H* and *LDOX*, which contribute to the accumulation of anthocyanin accumulation [35]. Further analysis on *WRKY* gene family found that *VvWRKY14*, *19* and *52* were highly expressed in berries during or following veraison, when berries begin to change color [36]. We also performed comparative proteomic analysis to investigate the complex protein variation in sunlight-exposed and sunlight-excluded grape berry skin, and found that the proteins involved in various functions were differentially accumulated [37]. These tools, including microarray and proteome analysis, digital gene expression profiles and analysis of the transcriptome, have proven invaluable in shedding light on the global changes in both gene and protein expression.

We previously reported a red grape ‘Jingyan’ *(V. vinifera*, red ‘Jingxiu’ × green ‘Xiangfei’) that is characterized by sunlight-independent anthocyanin accumulation in the berry skin, i.e. it can develop red coloring even when sunlight is excluded from the grape clusters from fruit set until maturity [38]. Using ‘Jingyan’ and its maternal parent ‘Jingxiu’, in which anthocyanin accumulation is sunlight-dependent and the berry skin fails to color if sunlight is excluded from the clusters, we found that *VvMYBA1* is differentially involved in anthocyanin biosynthesis in ‘Jingxiu’ and ‘Jingyan’, via the regulation of *VvUFGT* [38]. In this study, by taking advantage of digital gene expression profiling, which allows large-scale analysis of genetic variation and the profiling of many genes, we seek to further uncover the molecular events underlying the process of anthocyanin synthesis in response to light in these two contrasting cultivars.

## Results and Discussion

### Coloration and anthocyanin content

Grown under conditions of sunlight exclusion from fruit set (5 days after anthesis) to maturity, ‘Jingxiu’ clusters appeared green (Figure S1), and did not accumulate any anthocyanin at maturity (Table 1). In contrast, in the complete absence of sunlight, ‘Jingyan’ clusters still became red-colored, and accumulated a high total anthocyanin concentration (94.62 mg kg^−1^ FW in 2010 and 39.07 mg kg^−1^ FW in 2013), which were not significantly different from those under natural sunlight exposure (Table 1). This indicates that anthocyanin synthesis in ‘Jingxiu’ and ‘Jingyan’ berry skin was sunlight-dependent and -independent, respectively.

**Table 1:** Total anthocyanin concentration (mg/100 g fresh weigh) in berry skin for ‘Jingxiu’ and ‘Jingyan’ under conditions of sunlight exposure and sunlight exclusion in 2010 and 2013.

Year	‘Jingxiu’		‘Jingyan’	
	Sunlight exposure	Sunlight exclusion	Sunlight exposure	Sunlight exclusion
2010	45.50a	NDb	112.67	94.62
2013	70.26a	NDb	77.40	39.07

ND, non-detected.

Values followed by the different letter within a line for each cultivar in each year differ significantly at *P*<0.05 via *t*-test.

### Sequencing saturation analysis and unique tag alignment

A total of 9,570,351–10,843,174 tags were sequenced in this study (Table 2). There were 55,532 and 37,418 more unique tags in the sunlight-excluded berry skin libraries than in the sunlight-exposed berry skin libraries for ‘Jingxiu’ and ‘Jingyan’, respectively. These probably represent the gene response to sunlight exclusion. Unique tags with a copy number between 2 and 5 accounted for 59.25–62.20% of the total unique tags. The saturation of tags was evaluated based on the number of identified genes. When no new unique tags were detected, sequencing reached saturation (Figure S2). All the samples reached a plateau shortly after six M tags were sequenced. No new genes were identified as the tag number approached eight M. Since more than nine M available tags were generated in each sample, the tags were sequenced to saturation, producing a full representation of the transcripts in this study.

**Table 2:** Solexa tags in ‘Jingxiu’ and ‘Jingyan’ grape skins under conditions of sunlight exposure and sunlight exclusion.

	‘Jingxiu’		‘Jingyan’	
	Sunlight exposure	Sunlight exclusion	Sunlight exposure	Sunlight exclusion
Total tags	9 570 351	10 843 174	8 882 088	10 014 620
Clean tags	9 212 390	10 586 203	8 555 425	9 684 309
Clean tags copy number = 1	389 451	473 846	358 443	383 353
Unique tags	170 165	225 697	153 226	190 644
Copy number [2], [5]	102 253	141 278	90 781	116 559
Copy number [6], [10]	22 718	27 821	20 652	25 899
Copy number [11], [20]	15 348	19 113	14 056	16 681
Copy number [21], [50]	13 073	16 516	12 125	13 783
Copy number [51,100]	6 426	8 418	5 970	6 734
Copy number >100	10 347	12 551	9 642	10 988

The unique tags from each sample were compared against the published genome and gene sequences from ‘Pinot Noir’ (*V. vinifera*) in the Genoscope Grape Genome database (http://www.cns.fr/spip/Vitis-vinifera.html) using BLASTn. In total, 11,171 (42.40%, sunlight-exposed ‘Jingyan’) to 13,716 (52.06%, sunlight-excluded ‘Jingxiu’) genes were found, similar to those (47.81–50.51%) detected by the same Solexa sequencing technology in *V. amurensis* ‘Zuoshan-1′ [39]. Together, this indicates that a substantial proportion of the predicted transcripts were expressed in the grapes in this study.

### Identification and clustering analysis of differentially expressed genes (DEGs)

**Figure 1:**
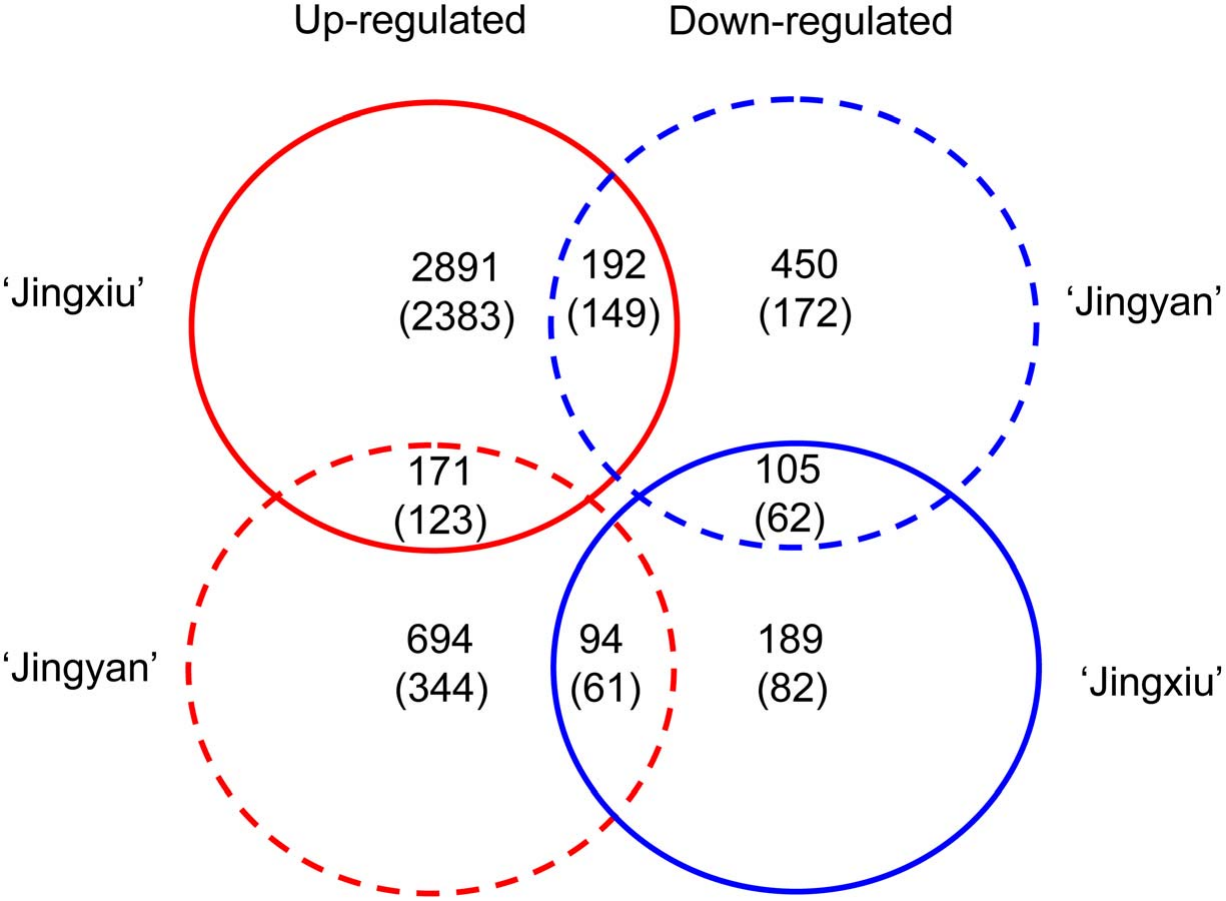
Numbers of differentially expressed genes with a log2 ratio≥1 at *P*<0.0001 in sunlight-excluded berry skin compared with sunlight-exposed berry skin for each cultivar. Solid- and dashed-line circles represent ‘Jingxiu’ and ‘Jingyan’, respectively. Red and blue circles represent up-regulated and down-regulated genes, respectively, relative to sunlight exposure. Numbers in parentheses indicate genes that exhibited a change in expression log2 ratio ≥2.

Unique tags that perfectly matched the reference genes in each sample were normalized to tags per million clean tags (TPM) and used to evaluate the expression level of transcripts. Only the genes that had more than 10 TPM in at least one of the sunlight-exposed and sunlight-excluded samples of each cultivar were considered further. A total of 3,642 and 1,706 genes, accounting for 14.7% and 3.5% of the genes in the *Vitis* genome (26,346), responded to sunlight exclusion in ‘Jingxiu’ and ‘Jingyan’ berry skin, respectively (Figure 1), indicating that the genomic response to sunlight exclusion in ‘Jingyan’ was less complex than in ‘Jingxiu’. Sunlight exclusion also resulted in more up-regulated genes–3,254 genes in ‘Jingxiu’ (89.3%) and 959 genes in ‘Jingyan’ (56.2%) – than down-regulated genes–388 genes in ‘Jingxiu’ (10.7%) and 747 genes in ‘Jingyan’ (43.8%).

**Figure 2:**
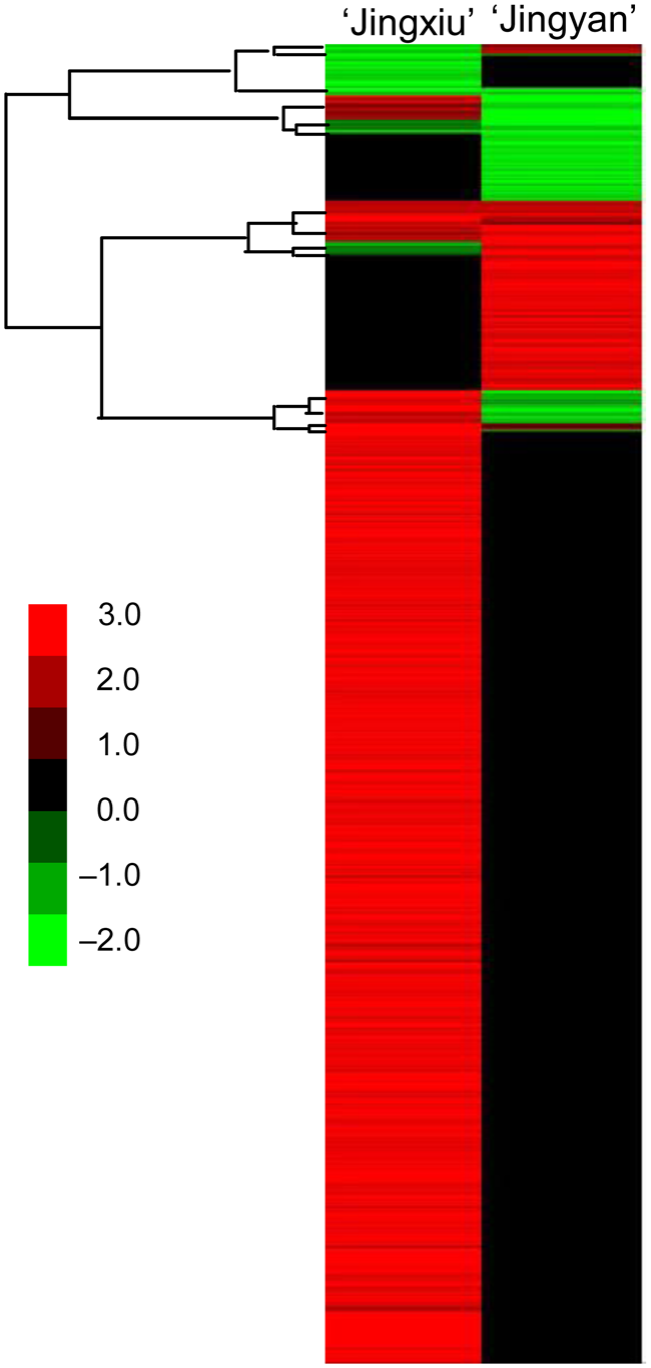
Standard average linkage (Euclidean distance) hierarchical clustering analysis of the differentially regulated genes that showed at least a twofold change (*P*<0.0001) between sunlight-excluded and sunlight-exposed berry skins of ‘Jingxiu’ and ‘Jingyan’ grapes. Different colors represent down- (–) and up- (+) regulated genes.

Restricting the observations to genes whose expression changed more than twofold between sunlight exposure and exclusion in either cultivar (Figure 1, numbers in parentheses), a total of 2,860 genes were regulated in response to sunlight exclusion in ‘Jingxiu’; of these, 2,655 genes were induced and 205 were repressed. Using the same twofold ratio as the cutoff criteria, sunlight exclusion resulted in 528 up-regulated and 383 down-regulated genes in ‘Jingyan’. As confirmed by average linkage hierarchical clustering analysis (Figure 2), there were distinct and overlapping groups of genes regulated in ‘Jingxiu’ and ‘Jingyan’. Some genes responded to sunlight exclusion in the same manner in both cultivars, of which 123 were up-regulated and 62 down-regulated. Other genes responded to sunlight exclusion in only one of the cultivars; specifically, 2,383 genes were up-regulated and 82 genes were down-regulated in ‘Jingxiu’ berry skin only, and 344 genes were up-regulated and 172 down-regulated in ‘Jingyan’ berry skin only. Some genes responded to sunlight exclusion in opposite ways in the two cultivars; 149 genes that were up-regulated in ‘Jingxiu’ were down-regulated in ‘Jingyan’, and 61 genes that were down-regulated in ‘Jingxiu’ were up-regulated in ‘Jingyan’.

### Function categories of differentially expressed genes (DEGs)

**Figure 3:**
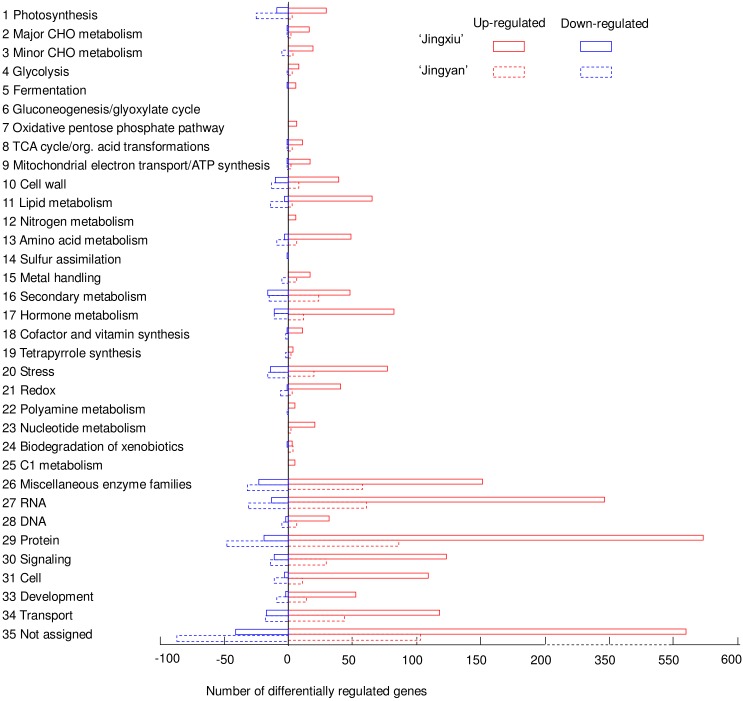
Numbers of down-regulated or up-regulated genes with log2 ratio≥2 at *P*<0.0001 between sunlight-excluded and sunlight-exposed berry skins of ‘Jingxiu’ and ‘Jingyan’ grapes, based on MapMan classifications.

By using MapMan functional categories, these genes were found to cover many functions, with the exception of 712 genes that were not assigned to any groups (Figure 3, Table S1). Protein and RNA categories accounted for the largest proportions (19.7% and 12.5%, respectively), probably due to their wider functional classification. Signaling, miscellaneous enzyme families and transport categories each accounted for 5.0–6.8%, while cell, stress, hormone, and lipid metabolism, as well as secondary metabolism categories, each accounted for 2–5%. The other categories accounted for less than 2% each. Full datasets are available online (Table S2). In the following sections, we mainly focus on: i) genes involved in flavonoid and phenylpropanoid metabolism, miscellaneous enzyme families, and transport categories that are related to anthocyanin synthesis and transport; ii) genes associated with the regulation of RNA transcription related to anthocyanin synthesis; and iii) genes involved in light signaling as well as protein degradation. All of these may be important elements in the relationship between sunlight and anthocyanin synthesis.

### Verification of digital gene expression by real-time PCR

Sixteen genes from six functional categories relating to our biological focus were selected to validate the digital gene expression profiles (year 2010) in a biologically independent experiment (year 2013) using quantitative real-time PCR (qRT-PCR). They included *CHS*, *UDPG*, *GST* (glutathione S-transferase) and *ANP* (anthocyanin permease) involved in anthocyanin synthesis and accumulation, three *MYB* genes in the regulation of anthocyanin synthesis, eight genes in ubiquitin-dependent protein degradation, and Cryptochrome 1 (*CRY1*) in the light signaling, which were more discussed in following sections. The qRT-PCR and digital gene expression results for these genes showed some differences in fold-changes, e.g. *UDPG* (GSVIVT01024419001) and *VvMYBA1* (GSVIVT01022659001) were down-regulated thirteen and five fold in sunlight-excluded ‘Jingxiu’ berry skin via the method of digital expression, respectively, while they were undetectable via qRT-PCR. It may be due to different conditions in the two years and differences in the sensitivity of the two methods [40], [41]. However, in general, qRT-PCR results showed good correlation and agreement with the digital expression data, thus verifying the method (Table 3).

**Table 3:** Digital expression and qRT-PCR analyses for a subset of genes.

ID	Annotation	Accession	E-value	Digital expression			qRT-PCR
				‘Jingxiu’	‘Jingyan’	‘Jingxiu’	‘Jingyan’
GSVIVT01032968001	Chalcone synthase [*Vitis vinifera*]	AAB72091.1	0.00E+00	–5	0	–3	–1
GSVIVT01024419001	UDP-glucose: flavonoid 3-O-glucosyltransferase [*Vitis vinifera*]	BAB41020.1	0.00E+00	–13	0	ND	–1
GSVIVT01035256001	Glutathione S-transferase [*Vitis amurensis*]	ACN38271.1	1.00E-120	–8	–1	–8	–1
GSVIVT01027811001	MYB transcription factor MYB4 [*Vitis vinifera*]	XP_002285193.1	0.00E+00	–2	1	–2	0
GSVIVT01027182001	MYBPA1 protein [*Vitis vinifera*]	NP_001268160.1	3.00E-169	7	0	4	1
GSVIVT01022659001	MYB-related transcription factor VvMYBA1 [*Vitis vinifera*]	BAD18977.1	0.00E+00	–5	–2	ND	–1
GSVIVT01016705001	COP1-interacting protein 7 (CIP7)-like protein [*Medicago truncatula*]	XP_003627371.1	0.00E+00	2	0	1	0
GSVIVT01009934001	COP1-interacting protein 8 (CIP8) isoform 1 [*Vitis vinifera*]	XP_002273623.1	2.00E-166	2	0	1	0
GSVIVT01030511001	E3 ubiquitin protein ligase COP1 [*Vitis vinifera*]	XP_002271415.1	0.00E+00	3	0	0	–1
GSVIVT01017738001	Cullin-1 isoform 1 [*Vitis vinifera*]	XP_002272195.1	0	3	0	7	0
GSVIVT01021502001	Cullin 4 [*Solanum lycopersicum*]	ABX09988.1	0.00E+00	2	0	1	0
GSVIVT01015070001	RING-box protein 1a [*Vitis vinifera*]	XP_002278332.1	5E-82	4	0	14	0
GSVIVT01003780001	COP9 signalosome complex subunit 4 (CSN4)-like [*Vitis vinifera*]	XP_003635093.1	3.00E-101	2	–2	1	0
GSVIVT01018273001	COP9 signalosome complex subunit 7 (CSN7) isoform 1 [*Vitis vinifera*]	XP_002273686.1	0.00E+00	7	0	8	0
GSVIVT01009033001	Cryptochrome 1 *[Vitis vinifera*]	ABX79355.1	0.00E+00	2	0	1	0
GSVIVT01028882001	Anthocyanin permease 1 [*Vitis vinifera*]	ACN91542.1	0.00E+00	–7	0	–4	–1

Data represent fold-change of genes down-regulated (–) or up-regulated (+) in sunlight-excluded and sunlight-exposed berry skins of ‘Jingxiu’ and ‘Jingyan’ grapes.

ND: the expression was not detectable in ‘Jingxiu’ berry skin under sunlight exclusion.

### DEGs in flavonoid and phenylpropanoid metabolism

**Figure 4:**
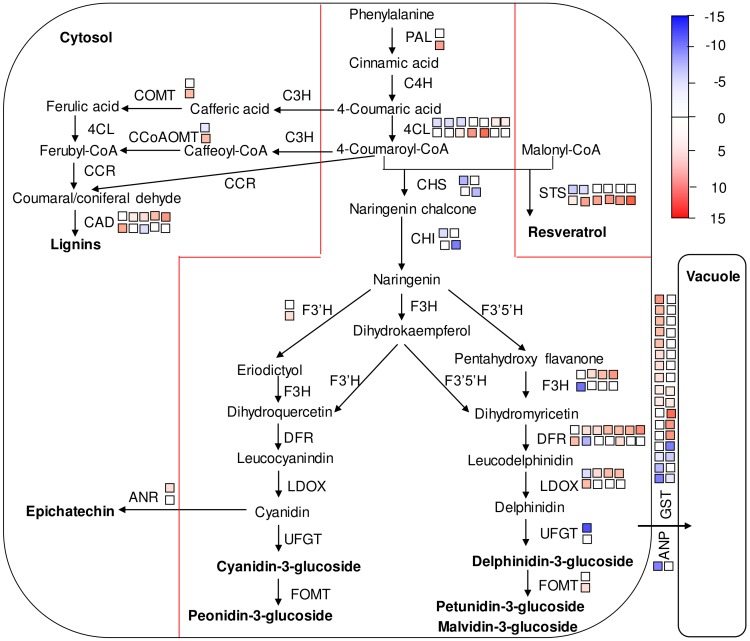
Overview of the phenylpropanoid pathway modulation. For each gene, the upper squares represent ‘Jingxiu’ and the lower squares ‘Jingyan’, while the number of squares represents the copy number of each gene. Different colors represent down-regulated or up-regulated genes in ‘Jingxiu’ and ‘Jingyan’ berry skin (sunlight exclusion versus exposure) with log2 ratio≥2 at *P*<0.0001.

The DEGs related to anthocyanin synthesis, transport in flavonoid and phenylpropanoid metabolism, miscellaneous enzyme families, and transport are schematically represented in Figure 4. Phenylalanine ammonia lyase (*PAL*) is the first step in the phenylpropanoid pathway and ensures flux through the general phenylpropanoid metabolism pathway in order to feed flavonoid, phenylpropanoid, and lignin biosynthesis [42]. This did not change in ‘Jingxiu’ berry skin after sunlight exclusion, but it was up-regulated slightly in ‘Jingyan’ berry skin. 4-coumarate: CoA ligase (*4CL*), *CHS*, *CHI*, *F3H*, *DFR*, and *LDOX* each had several copies, and the copies of each gene responded differently to sunlight exclusion in the two cultivars. UFGT, which catalyzes the final step of color accumulation, correlates at the transcript level with the accumulation of anthocyanins, and is widely considered the key enzyme determining coloration in grape berry skin [43], [44]. In this study, *UFGT* expression was down-regulated 13-fold (and undetectable via qRT-PCR) in ‘Jingxiu’ after sunlight exclusion, but remained almost unchanged in ‘Jingyan’. Recently, there has been increasing evidence to suggest that *AOMT* expression correlates with the accumulation of methylated anthocyanins in grapevines [45], [46]. The up-regulation of anthocyanin-O-methyltransferase (*AOMT*) expression in ‘Jingyan’ after sunlight exclusion would therefore promote anthocyanin accumulation.

Stilbenes and lignins represent branching points in the phenylpropanoid pathway. *STS* channels 4-coumaroyl-CoA molecules towards stilbene synthesis. Caffeic acid 3-O-methyltransferase (COMT) and caffeoyl-CoA-O-methyltransferase (CCoAOMT) are key enzymes in the process of lignin synthesis [47]–[49]. The six *STS*s detected, *COMT* and *CCoAOMT* were all up-regulated under sunlight exclusion in ‘Jingyan’, but were either down-regulated or unchanged in ‘Jingxiu’. Although stilbene and lignin biosynthesis competes for the precursor phenylalanine with anthocyanin biosynthesis, the up-regulated *PAL* probably enhanced anthocyanin biosynthesis, as well as stilbene and lignin biosynthesis, in sunlight-excluded ‘Jingyan’ berry skin. In sunlight-excluded ‘Jingxiu’ berry skin, however, stilbene and lignin biosynthesis may either be suppressed or not affected.

### GST and ANP

GST is a large, complex gene family best known for their ability to catalyze the conjugation of the reduced form of glutathione to xenobiotic substrates for the purpose of detoxification. Sixty-four of the 87 predicted *GST*s in grapevines were detected during berry development of the grape ‘Corvina’ using RNA-seq analysis [50]. However, the specific roles of the individual *GST*s were not clear. It was reported that there was strong correlation between *GST* expression and the accumulation of anthocyanin in *V. vinifera* cell cultures [51], as well as in ‘Norton’ and ‘Cabernet Sauvignon’ berry skin [52]. ANP is an anthocyanin vacuolar transporter, and has been suggested to participate in anthocyanin vacuolar sequestration [53], [54].

In this study, 16 *GST*s were detected and differentially regulated by sunlight exclusion in the two cultivars (Figure 4). Among them, a grapevine *GST* (GSVIVT01035256001), identified as one of the most responsive *GST*s that increase anthocyanin accumulation [51], was selected for qRT-PCR analysis. The transcript level of this *GST* gene was down-regulated eightfold in sunlight-excluded ‘Jingxiu’, but down-regulated only onefold in sunlight-excluded ‘Jingyan’. We speculate that the almost unchanged transcript levels of this *GST* in sunlight-excluded ‘Jingyan’ skin may contribute to anthocyanin accumulation in the vacuoles. However, the participation of the 16 *GST*s we detected in the compartmentalization of anthocyanins in the vacuole requires further experimental verification.

An *ANP* gene (GSVIVT01028882001) was identified in this study (Figure 4). The encoded protein had 67.4% similarity to an anthocyanin permease which was up-regulated in *ANT1*-tomato [53] and 85.9% similarity to *anthoMATE*, a gene that is thought to be involved in vacuolar anthocyanin transport in *V. vinifera* [54]. Using qRT-PCR, the transcript levels of the *ANP* gene were found to be almost unchanged in sunlight-excluded ‘Jingyan’, but down-regulated sevenfold in sunlight-excluded ‘Jingxiu’. It is yet to be determined if the difference at the transcript level of this particular *ANP* could result in the differential production of anthocyanins after sunlight exclusion.

Although the *ANP* and *GST* genes are not directly related to the anthocyanin biosynthetic pathway, *GST* and *ANP* remain the most relevant candidates for the differential regulation of the response of the two cultivars to sunlight exclusion.

### Transcription factors MYB, bHLH, and WD40

Although the WD40-repeat family proteins were not specifically assigned to any functional categories by MapMan, they are implicated in a variety of functions, such as signal transduction and transcription regulation. In this study, we consider them, together with MYB and bHLH, as transcription factors, because the bHLH and WD40 families are known to play a role in the functioning of the *MYB* family in the regulation of anthocyanin synthesis [21], [22]. A set of 26 *MYB*, 14 *bHLH* and 23 *WD40* genes were found to respond differently to sunlight exclusion in the two cultivars (Table 4).

**Table 4:** Numbers of down-regulated or up-regulated genes in some MapMan classifications, which had log2 ratio≥2 at *P*<0.0001, in sunlight-excluded and sunlight-exposed berry skins of ‘Jingxiu’ and ‘Jingyan’ grapes.

Bin	Bin name	Xup	Xup-Yup	Xup-Ydown	Xdown	Xdown-Yup	Xdown-Ydown	Yup	Ydown	Total
27.3.6	RNA.regulation oftranscription.bHLH, BasicHelix-Loop-Helix family	10	1			1		1		13
27.3.25	RNA.regulation oftranscription.MYB domaintranscription factor family	12			3	2	2	7		26
35.1	not assigned.noontology-WD(40) repeat	10	1					3		14
										
29.5.11.1	protein.degradation.ubiquitin.ubiquitin	9				1				10
29.5.11.2	protein.degradation.ubiquitin.E1	2								2
29.5.11.3	protein.degradation.ubiquitin.E2	20	3					1	1	25
29.5.11.4.1	protein.degradation.ubiquitin.E3.HECT	3	1							4
29.5.11.4.2	protein.degradation.ubiquitin.E3.RING	65	2	2				7	4	80
29.5.11.4.3.1	protein.degradation.ubiquitin.E3.SCF.SKP	1								1
29.5.11.4.3.2	protein.degradation.ubiquitin.E3.SCF.FBOX	27	1	3	1			4	1	37
29.5.11.4.3.3	protein.degradation.ubiquitin.E3.SCF.cullin	5								5
29.5.11.4.5	protein.degradation.ubiquitin.E3.BTB/POZ Cullin3	3								3
29.5.11.5	protein.degradation.ubiquitin.ubiquitin protease	5						1		6
29.5.11.20	protein.degradation.ubiquitin.proteasom	21	1							22
										
30.11	Signalling.light	6		1			1	1	1	10

Xup-Yup and Xdown-Ydown indicate that genes were, respectively, up-regulated and down-regulated in both ‘Jingxiu’ and ‘Jingyan’; Xup, Xdown, Yup, Ydown indicate that genes were up-regulated and down-regulated in either ‘Jingxiu’ or ‘Jingyan’; Xup-Ydown and Xdown-Yup indicate that genes were regulated contrarily in ‘Jingxiu’ and ‘Jingyan’.

A homology analysis of *V. Vinifera* genes was conducted, and three *MYB* transcription factor genes, *VvMYBA1* (GSVIVT01022659001), *VvMYB5b* (GSVIVT01025452001), and *VvMYBPA1* (GSVIVT01027182001) were found to be associated with anthocyanin synthesis in grape berry skin, while *VvMYBA3* (GSVIVT01022664001) played no role [55]. In grapes, *VvMYBA1* is widely considered to be involved in the regulation of *VvUFGT* [56]. *VvMYBA1* was down-regulated fivefold in sunlight-excluded ‘Jingxiu’ berry skin (and undetectable with qRT-PCR), which is consistent with the finding for ‘Cabernet Sauvignon’ grapes [11]. However, in sunlight-excluded ‘Jingyan’ berry skin, *VvMYBA1* was down-regulated much less (approximately twofold), which still resulted in sufficient expression levels to promote *UFGT* expression and phenotypic coloration [38]. Both *VvMYB5b* and *VvMYBPA1* were up-regulated in sunlight-excluded ‘Jingxiu’, but remained unchanged in sunlight-excluded ‘Jingyan’. *VvMYB5b* induces several flavonoid biosynthesis steps, including anthocyanins, condensed tannins, flavonols, and lignin [57], while *VvMYBPA1* regulates the final steps of proanthocyanidin production [58], [59]. The precise functions of *VvMYB5b*, *VvMYBPA1* and the other *MYB*, *bHLH* and *WD40* transcription factor genes in this study are currently unknown.

### DEGs in ubiquitin-dependent protein degradation

Protein degradation via the ubiquitin pathway plays an essential role in diverse cellular pathways such as cell-cycle progression, DNA repair, endocytosis, and apoptosis, as well as in signal transduction. In this study, 189 genes were classified as being involved in ubiquitin-dependent protein degradation (Table 4). None of these were down-regulated in ‘Jingxiu’ in response to sunlight exclusion; 166 were up-regulated, while the other 23 genes did not change. In contrast, the majority (162) remained unchanged in ‘Jingyan’ berry skin in response to sunlight exclusion, nine were down-regulated, and 18 were up-regulated. Thus, overall, proteins were more strongly degraded in ‘Jingxiu’ than in ‘Jingyan’ as a result of sunlight exclusion.

Ubiquitin is conjugated to target proteins through the sequential actions of the ubiquitin-activating enzyme (E1), ubiquitin-conjugating enzymes (E2), and ubiquitin-protein ligases (E3), and several mutants in light signaling have been mapped to this pathway in *Arabidopsis* [60]. Among the E3 genes, *COP1* (constitutively photomorphogenic 1, GSVIVT01030511001) is one crucial transcription factor in the light signal transduction system, whose functions have been studied most in *Arabidopsis.* This gene plays an important role in plant development that is induced by light signaling, for example, as a master repressor of photomorphogenesis, which includes anthocyanin accumulation [61], [62]. *COP1* was differentially regulated by sunlight exclusion in the two cultivars; specifically, it was increased threefold in ‘Jingxiu’ and was unchanged in ‘Jingyan’. Its up-regulation in sunlight-excluded ‘Jingxiu’ enhances the suppression of anthocyanin accumulation, whereas sunlight-excluded ‘Jingyan’ can synthesize anthocyanins normally. COP1 activity is mediated by several protein complexes, for example, COP9 signalosome (CSN), cullins (CUL), damaged DNA-Binding protein 1 (DDB1), RING-Box 1 (RBX1), and suppressor of PhyA (SPA) proteins [63], as well as COP1-interacting proteins (CIP) [64]. Accordingly, it is not surprising that five cullins, including three BTB/POZ domain *Cul3* (GSVIVT01008796001, GSVIVT01010205001, GSVIVT01003474001), *Cul1* (GSVIVT01017738001) and *Cul4* (GSVIVT01021502001), two *CIP*s (*CIP7*, GSVIVT01016705001; *CIP8*, GSVIVT01009934001), a *RBX1* (GSVIVT01015070001), and two *CSN*s (*CSN4*, GSVIVT01003780001; *CSN7*, GSVIVT01018273001), which were placed in the light signal category in MapMan (Table S2; Table 4), were induced in ‘Jingxiu’ by sunlight exclusion, while *CSN4* was suppressed and the others were unchanged in ‘Jingyan’. In addition, HY5 is believed to be one of the positive central modulators for the coordination of light signaling and the regulation of anthocyanin-associated gene expression [61], [65], and is targeted by COP1 for degradation in the absence of light. In this study, *HY5* was not differentially expressed in either cultivar at the transcript level; it therefore may function at the protein level instead.

The function of the proteasome is to degrade extraneous or damaged proteins by proteolytic reactions carried out by enzymes called proteases. According to the findings for *Arabidopsis*, suppression of photomorphogenesis is involved in ubiquitin-proteasome-mediated degradation of light-induced factors [64]. We found 22 proteasomal genes and four protease genes which were induced in sunlight-excluded ‘Jingxiu’ but remained unchanged in sunlight-excluded ‘Jingyan’. It would be interesting to investigate protein degradation via the ubiquitin pathway with respect to the mechanisms underlying sunlight-dependent versus -independent anthocyanin synthesis.

### DEGs in light signaling

Light is sensed by plants via several classes of photoreceptors that include the red and far-red light-sensing phytochromes, the blue/ultraviolet (UV)-A-perceiving cryptochromes and phototropins, and the UV-B-sensing photoreceptor UVR8 [66]. Cryptochromes, phototropins [67], and UVR8 are known to be involved in anthocyanin biosynthesis in plants [68]. However, with the exception of the two *CSN*s discussed above, we found only eight genes in the light signal capture system in which these photoreceptors are involved to be altered by sunlight exclusion in either or both cultivars (Table S2; Table 4). This indicates that the alteration of the gene expression profiles by sunlight exclusion may primarily occur in the transduction system downstream of the photoreceptors.

The eight genes responded differently to sunlight exclusion in the two cultivars. We identified three far-red-impaired response (FAR1)-related sequences (*FRS4*, GSVIVT01029436001; *FRS9*, GSVIVT01023847001; *FRS11*, GSVIVT01035045001) that are positive regulators essential for phytochrome A-controlled far-red responses in *Arabidopsis*. *FRS4* and *FRS9* were up-regulated by sunlight exclusion in ‘Jingxiu’, while *FRS11* was up-regulated in ‘Jingyan’. *CRY1* (GSVIVT01009033001), phytochrome, flowering time 1 (*PFT1*, GSVIVT01011939001, which acts in the phyB pathway and induces flowering in response to suboptimal light conditions; [69]) and negatively light-regulated gene (GSVIVT01030913001, which increases twentyfold after 48 h of light exclusion in mature *Arabidopsis thaliana* plants; [70]) were up-regulated two- to nine-fold by sunlight exclusion in ‘Jingxiu’, but remained unchanged in ‘Jingyan’.

Early light-induced genes (*ELIP*s, GSVIVT01018044001) were down-regulated by sunlight exclusion in both ‘Jingxiu’ (fourfold) and ‘Jingyan’ (fivefold). This is consistent with the fact that ELIPs accumulate almost linearly with increasing light intensities and are involved in the protection of the photosynthetic apparatus [71]. They are also suppressed by the dark [72]. Root phototropism protein 2 (RPT2) transduces signals downstream of phototropins to induce the phototropic response. Furthermore, RPT2 is a signal transducer involved in the phototropic response and stomatal opening, by association with phototropin 1, in *Arabidopsis* [73]. This gene (GSVIVT01024542001) was down-regulated threefold in ‘Jingyan’. The question of whether these genes play key roles in the different responses of the two cultivars to sunlight exclusion requires further investigation.

## Conclusions

Global gene expression by Solexa-based sequence was analyzed in the berry skin of two red grape cultivars, which can (‘Jingyan’) or cannot (‘Jingxiu’) synthesize anthocyanins after sunlight exclusion from fruit set until maturity. Some genes/pathways, such as *AOMT*, *GST*, *ANP*, *MYB*, *bHLH* and *WD40* families as well as ubiquitin-dependent protein degradation (e.g. COP9 signalosome, cullins, RING-Box 1, COP1-interacting proteins) were found to be interesting for further study. This study provides a valuable overview of the genetic background that may be responsible for sunlight-dependent versus -independent anthocyanin biosynthesis in berry skin.

## Methods

### Plant material and treatment

The red grapes ‘Jingxiu’ and ‘Jingyan’ (*V. Vinifera*) were obtained from the experimental vineyard of the Institute of Botany, Chinese Academy of Sciences, Beijing, in 2010 and 2013. ‘Jingyan’ is an offspring of ‘Jingxiu’ × ‘Xiangfei’ (*V. vinifera,* green). Both ‘Jingxiu’ and ‘Jingyan’ are early-ripening cultivars and in Beijing usually mature in late July and early August, respectively. The vines, grafted on ‘Beta’ rootstocks, were planted in a 4 m-high rainproof plastic shelter in 2005. The vines were spaced 1.5 m apart within the rows and 2.5 m apart between rows, with a north-south row orientation, and were trained to cordons. The entire vineyard was managed under the same conditions with respect to fertilization, irrigation, pruning, and disease control.

Six vines per cultivar were selected based on the uniformity of shoot growth and cluster development, and shoots were thinned to one cluster at fruit set. Two treatments were applied to clusters of each cultivar: sunlight exposure and sunlight exclusion. For sunlight exposure, the clusters were exposed to full sunlight throughout the growing season. Sunlight exclusion commenced when the berry diameter was approximately 2 mm, five days after anthesis. Clusters were placed inside an opaque box [74], until maturity. Light transmission through the box was zero for UV, visible, and IR light, according to the Spectrum Transmission Meter (LS108, Linshang, Shenzhen, China), and was less than 0.01% in the wavelength range 350–1100 nm, as measured by a photometer (Specord 200, AnalytikJena, Jena, Germany). Quantum light sensors (LI-COR LI 6400, Lincoln, NE, USA) were placed inside the box in the same manner as the clusters, and the level of photosynthetically active radiation (PAR) inside the box was observed to be less than 0.25% of that outside the box in the range 1000–2000 mmol m^–2 ^s^–1^. The temperature surrounding the clusters was monitored with data loggers (ZDR-20h, Zeda, Hangzhou, China) and the temperature difference between inside and outside the box was within 0–2°C, under ambient canopy temperatures ranging from 16–42°C.

Three replicates of two clusters each were randomly sampled at maturity in 2011, and four replicates were examined in 2013. Berry maturity was determined based on the seed color changing to dark brown without any senescence of berry tissue, and in reference to maturity date records from previous years. All the grape berry samples were peeled with forceps. The cleaned skin was immediately frozen in liquid N_2_ and stored at –80°C. The frozen skins sampled in 2011 were used for digital gene expression library construction, and those sampled in 2013 were subjected to qRT-PCR analysis.

### Anthocyanins analysis

Anthocyanin analysis was determined by HPLC-MS/MS [38].

### Construction of the digital gene expression library, and Solexa sequencing

Total RNA was isolated from the pooled samples of three replicates using the Plant Total RNA isolation kit (Tiandz Inc., Beijing, China). The Gene Expression Sample Prep Kit (Illumina Inc., San Diego, CA, USA) was used for sequence tag preparation, according to the manufacturer’s protocol. Six micrograms of total RNA were extracted and the mRNA was purified via Biotin-Oligo(dT) magnetic bead adsorption.

First strand cDNA synthesis was performed with oligo(dT) on the beads. After second-strand cDNA synthesis, double-stranded cDNA was digested with NlaIII endonuclease to produce a bead-bound cDNA fragment containing a sequence from the 3′-most CATG to the poly(A) tail. These 3′ cDNA fragments were purified using magnetic bead precipitation, and Illumina adapter 1 (GEX adapter 1) was added to the newly formed 5′ sticky end of the cDNA fragments. The junction of the GEX adapter 1 and CATG site was recognized by *MmeI*, a type I endonuclease (with separate recognition sites and digestion sites). The enzyme cuts 17 bp downstream of the CATG site, producing 17 bp cDNA sequence tags with GEX adapter 1. After removing the 3′ fragments by magnetic bead precipitation, Illumina adapter 2 (GEX adapter 2) was ligated to the new 3′ end of the cDNA fragments. These cDNA fragments represented the tag library.

A linear PCR amplification with 15 cycles was performed with primers complementary to the adapter sequences, to enrich the samples with the desired fragments, using Phusion polymerase (Finnzymes, Espoo, Finland). The resultant 85 base strips were purified by 6% TBE-PAGE gel electrophoresis. These strips were then digested and the single chain molecules were fixed onto the Solexa Sequencing Chip (flow cell). Each molecule grew into a single-molecule cluster sequencing template through *in situ* amplification, which represented a single tag derived from a single transcript. Four color-labeled nucleotides were added, and sequencing was performed using the Illumina HiSeq 2000 System (Beijing Genomics Institute, BGI, www.genomics.org.cn). The resultant 49 bp sequences contain target tags and 3′adaptor. Base-calling was performed using Illumina Pipeline. After purity filtering and initial quality tests, the reads were sorted and counted for the following analysis.

### Sequence annotation

‘Clean Tags’ were obtained by trimming adapter sequences and filtering adaptor-only tags and low-quality tags (containing ambiguous bases), using the Fastx-toolkit (http://hannonlab.cshl.edu/fastx_toolkit). Sequence alignment was done with Bowtie 0.12.8 using the Genoscope Grape Genome database (http://www.genoscope.cns.fr/externe/GenomeBrowser/Vitis/). The VBI microbial database (http://vmd.vbi.vt.edu/) and the BROAD institute database (http://www.braodinstitute.org/scientific-community/data) were used to exclude any tags contaminated by viruses. All clean tags were annotated based on transcript sequences of grape reference genes, masked grape genome sequences (excluding the repeating sequences) and NCBI. For conservative and precise annotation, only sequences with perfect homology or one nt mismatch were considered for the further annotation.

### Identification of differentially expressed genes

Numbers of annotated clean tags for each gene were calculated after alignment and then normalized to TPM (tags per million clean tags) [75], [76]. The genes that had less than 10 TPM in both the sunlight exposure and sunlight exclusion libraries for each cultivar were excluded first. The default value (tag number) of genes that were not found in one of the libraries was one. Differentially expressed genes (DEGs) in sunlight-excluded berry skins compared with sunlight-exposed berry skin for each cultivar were identified based on a rigorous algorithm [77]. *P* value was used to test the authenticity of differential transcript accumulation [77]. The Bonferroni corrected *P*-value was applied to control the FDR (false discovery rate) in the multiple comparison and analysis during the identification of DEGs [78]. An ‘FDR<0.001 and the absolute value of log2 ratio≥1′ was used as the threshold to determine the significance of gene expression differences. The transcripts with at least a twofold difference between the sunlight exposure and exclusion libraries for each cultivar (FDR<0.001) were assigned to functional categories using MapMan (http://mapman.gabipd.org/web/guest/mapmanstore, Vvinifera_145).

### Real-time PCR analysis

Total RNA was isolated from berry skin sampled in 2013 using the Universal Plant Total RNA Extraction Kit (Bioteke Corporation, Beijing, China). For synthesis of cDNA, 500 ng high-quality total RNA was treated with 5×DNA Buffer to remove DNA contamination.

First-strand cDNA was synthesized using RT Enzyme Mix, 10× Fast RT Buffer, and FQ-RT Primer Mix, according to the manufacturer’s instructions (Tiangen Biotech, Beijing, China). qRT-PCR was run with 5× diluted cDNA, gene-specific primers (Table S3), and SYBR Green Real MasterMix (Tiangen Biotech, Beijing, China) using a Mx3000P Real-Time PCR system (Stratagene, La Jolla, CA, USA). Thermal cycling conditions were 94°C for 2 min, followed by 40 cycles of 94°C for 10 s, 58°C for 18 s, and 68°C for 20 s. Fluorescent signals were recorded at the end of each cycle and a melting curve analysis was performed from 68–95°C.

Transcript levels were normalized against the average of the grapevine reference genes *VvUbiquitin 1* (BN000705). Analyses of qRT-PCR data used the 2^−ΔΔCT^ method. ΔC_T_ is equal to the difference in threshold cycles for the target (X) and reference (R) (C_T,X_–C_T,R_) genes, while ΔΔCT is equal to the difference of ΔC_T_ for the control (C) and treatment (T) (C_T,T_–C_T,C_) groups [79]. Experiments were performed with four biological replicates and three technical replicates. Reaction specificities were tested with melting gradient dissociation curves, electrophoresis gels, and cloning and sequencing of each PCR product.

## Supporting Information

Figure S1 **‘Jingxiu’ and ‘Jingyan’ grape clusters at maturity.**
(DOC)

Figure S2 **Accumulation of Solexa total tags in ‘Jingxiu’ and ‘Jingyan’ grape skins.**
(DOC)

Table S1 **Numbers of down-regulated or up-regulated genes with log2 ratio ≥2 in grape berry skin.**
(DOC)

Table S2 **List of differentially expressed genes with Log2Ratio > = 2 in grape berry skin.**
(XLS)

Table S3 **Forward (F) and reverse (R) primers and expected amplicon sizes of genes for qRT-PCR.**
(DOC)
