# Supplementary material for: Genome-Wide Transcriptional Profiles of the Berry Skin of Two Red Grape Cultivars (Vitis vinifera) in Which Anthocyanin Synthesis Is Sunlight-Dependent or -Independent
Source: PLoS One. 2014 Aug 26;9(8):e105959. doi: 10.1371/journal.pone.0105959 (PMC4144973; doi:10.1371/journal.pone.0105959)
Supplement: Figure S1 — ‘Jingxiu’ and ‘Jingyan’ grape clusters at maturity. (DOC) [file pone.0105959.s001.doc]

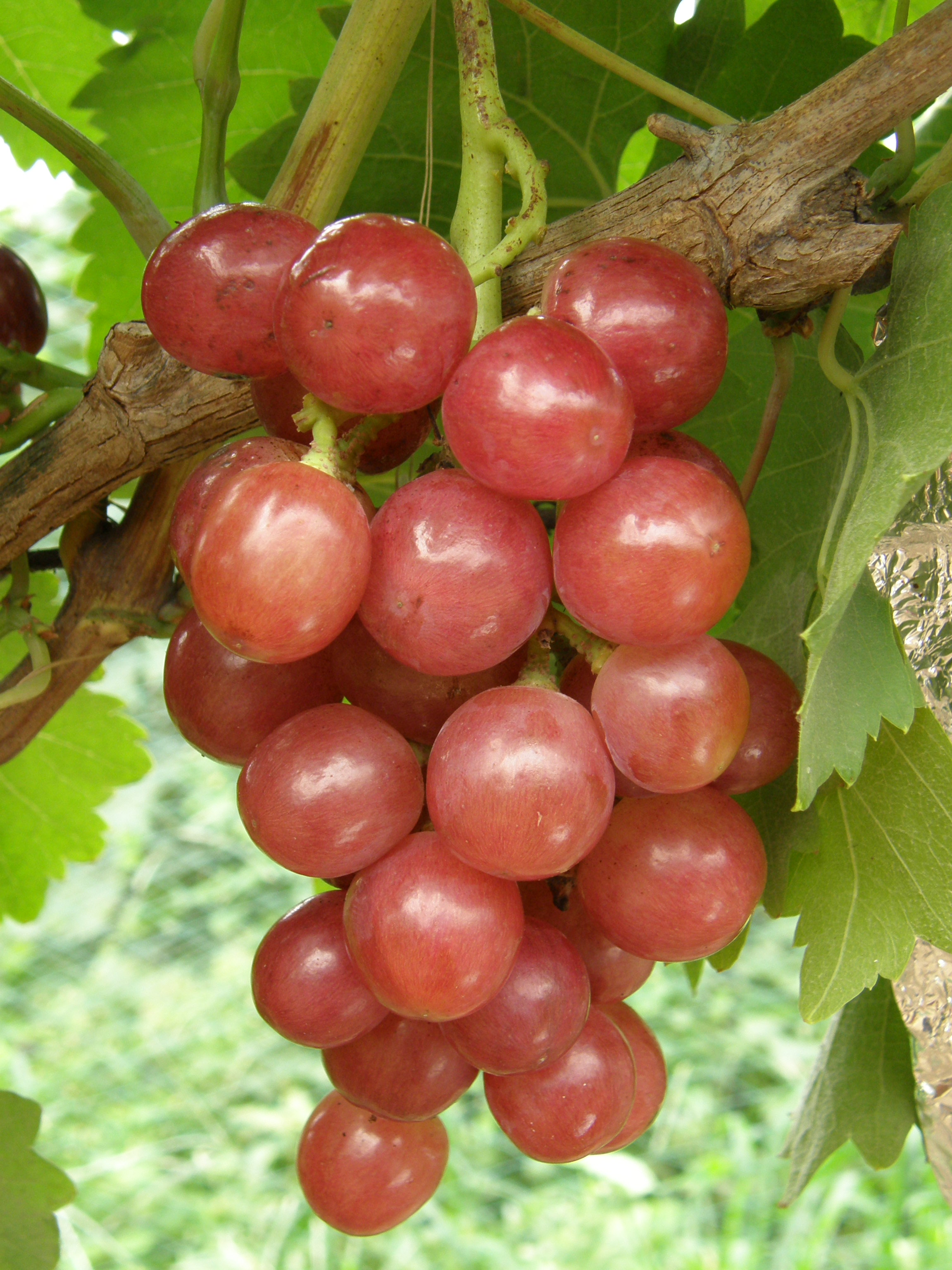

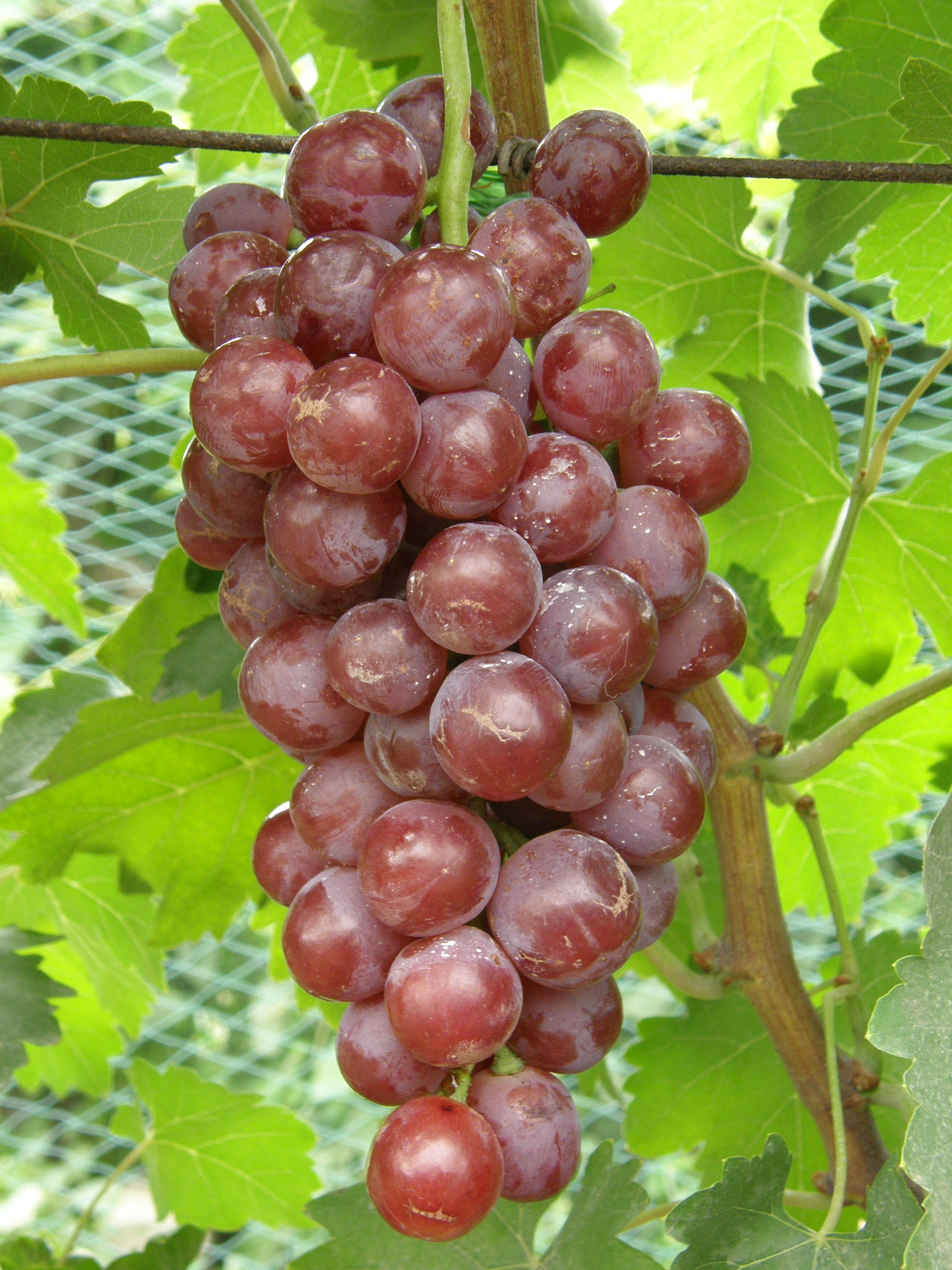

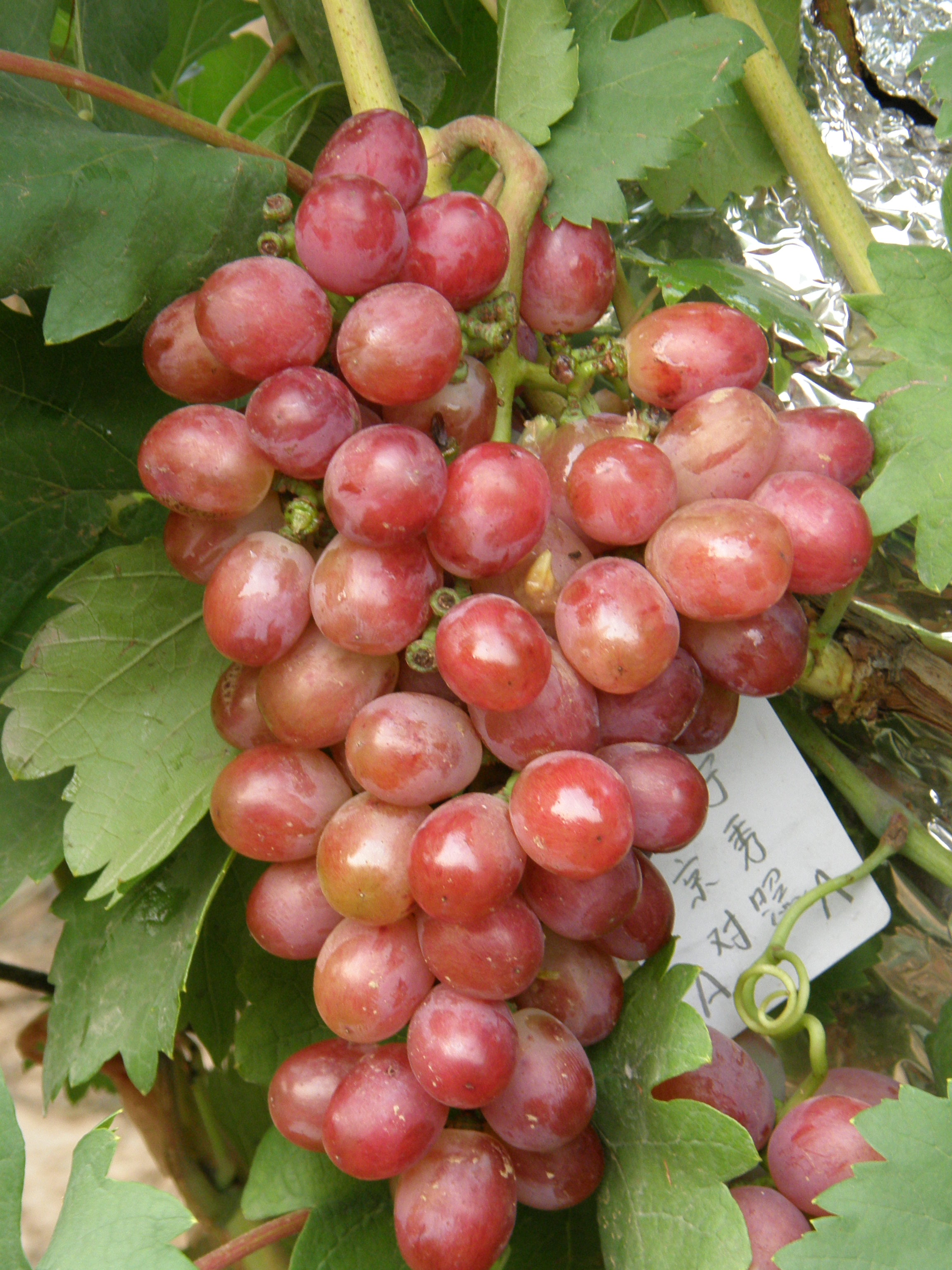

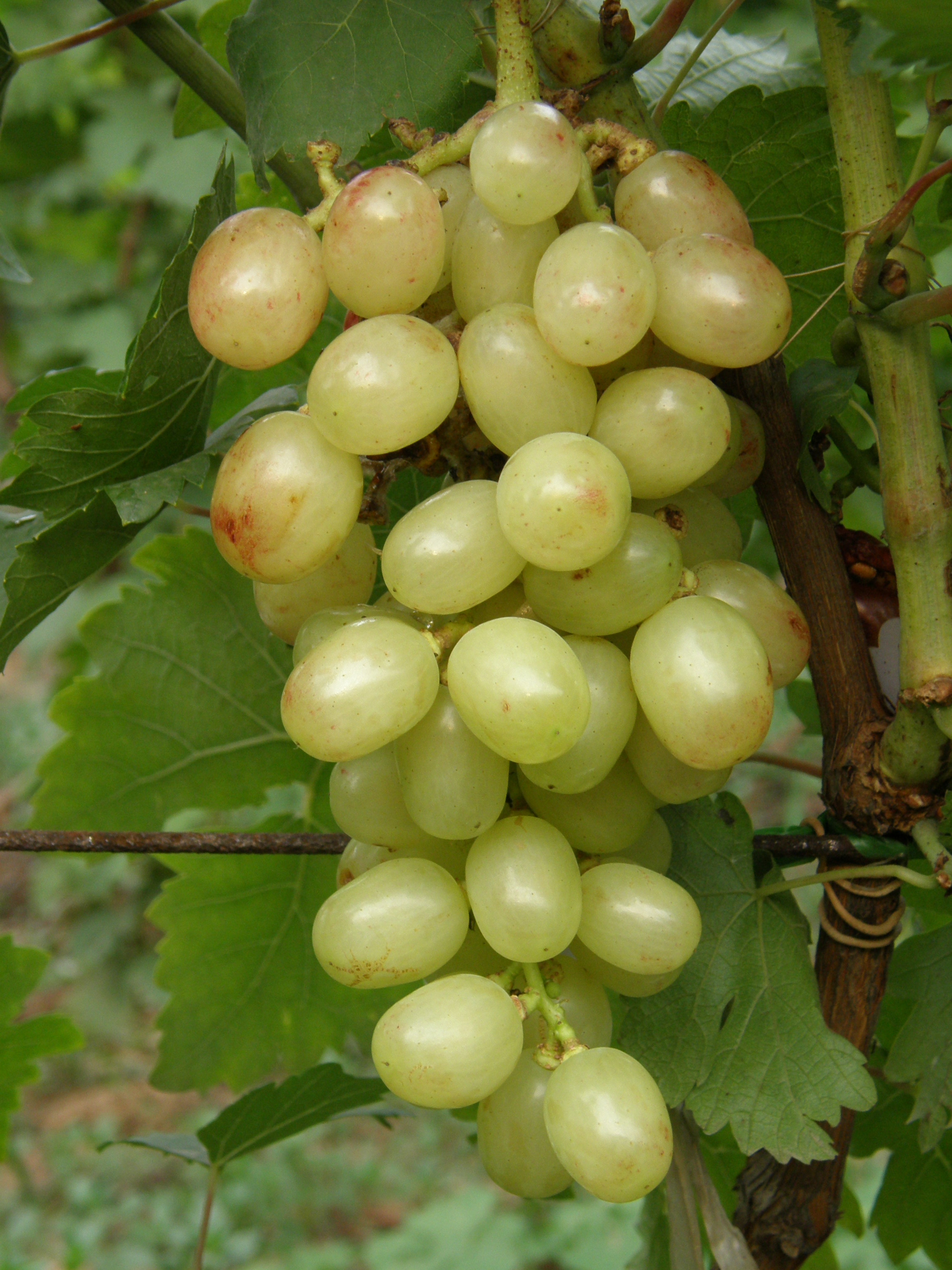

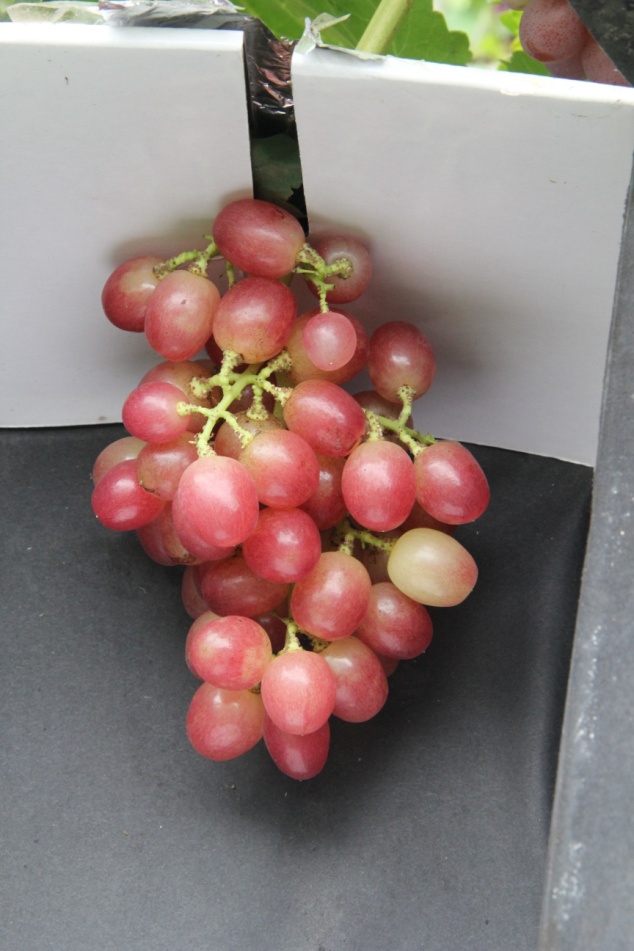

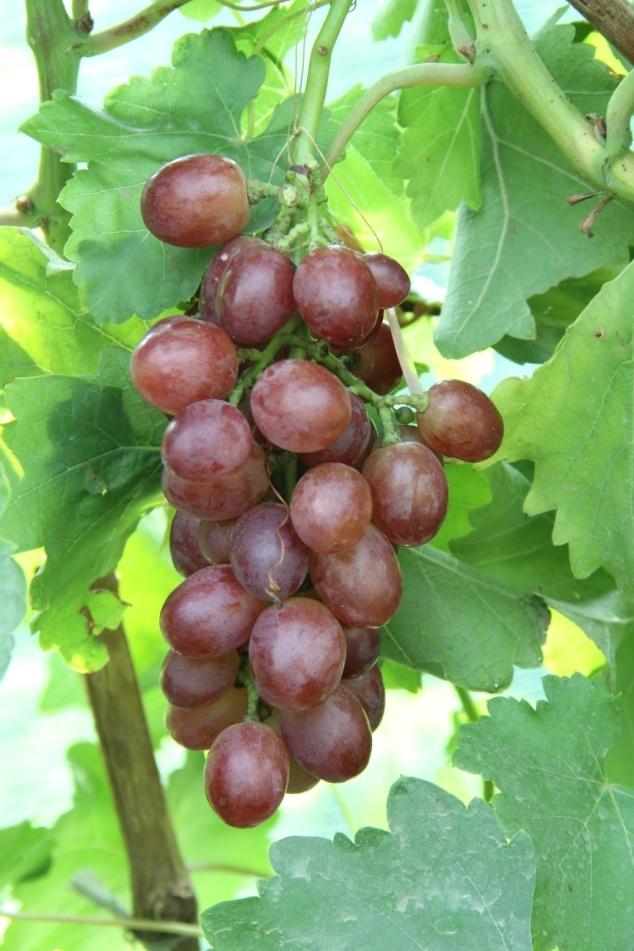

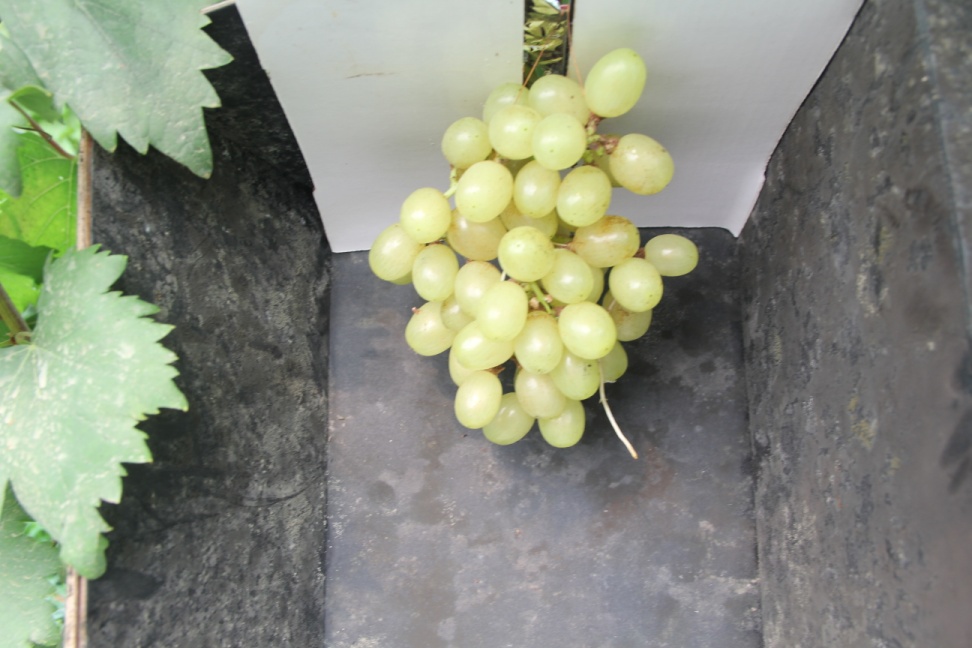

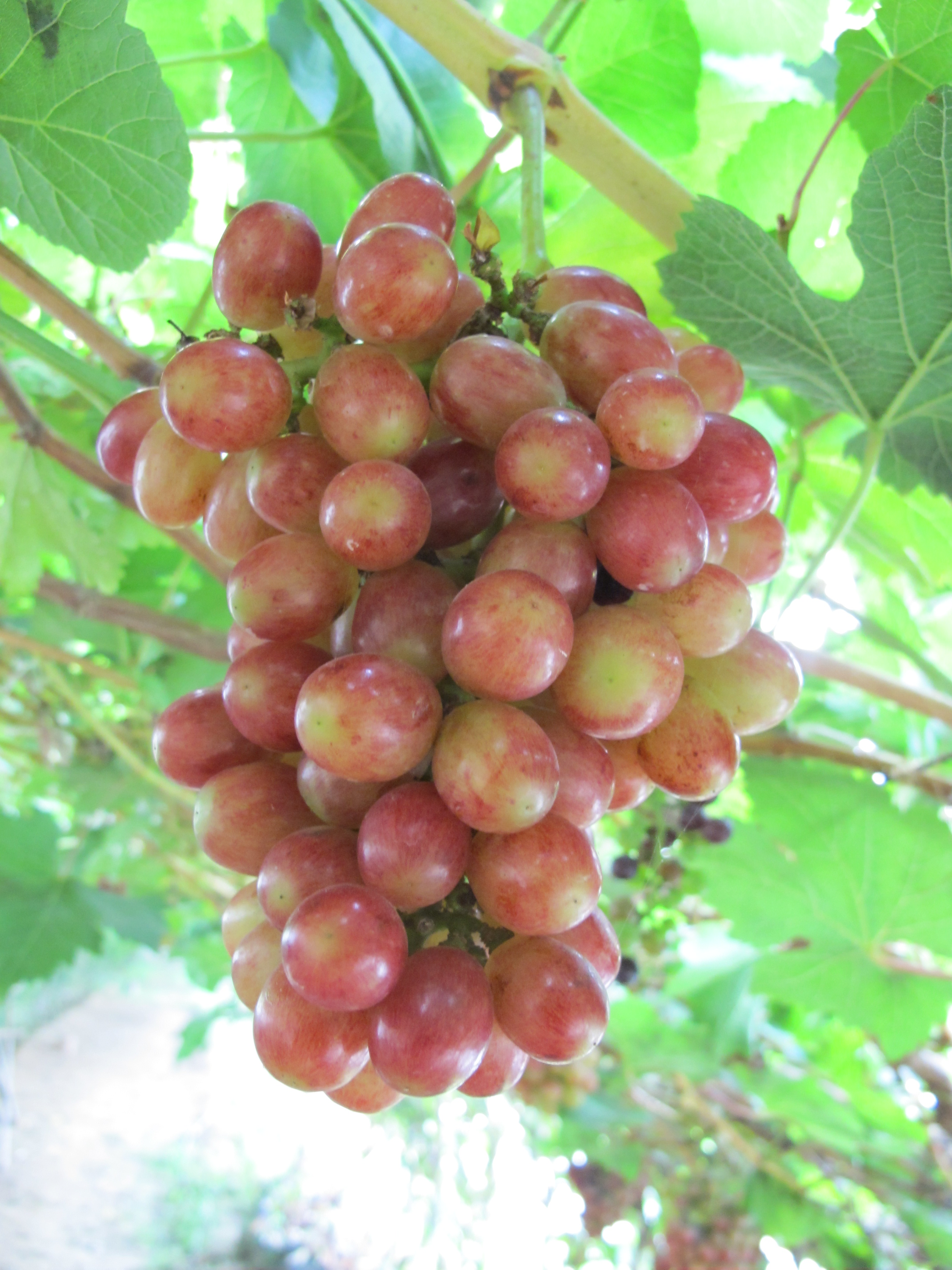


2010

2013

‘Jingxiu’

‘Jingyan’

Sunlight exposure

Sunlight exclusion

Sunlight exposure

Sunlight exclusion

**Figure S1.** ‘Jingxiu’ and ‘Jingyan’ grape clusters at maturity, following sunlight exclusion from five days after anthesis to maturity, or sunlight exposure throughout berry development, in 2010 and 2013.
