# Supplementary material for: Genome-Wide Transcriptional Profiles of the Berry Skin of Two Red Grape Cultivars (Vitis vinifera) in Which Anthocyanin Synthesis Is Sunlight-Dependent or -Independent
Source: PLoS One. 2014 Aug 26;9(8):e105959. doi: 10.1371/journal.pone.0105959 (PMC4144973; doi:10.1371/journal.pone.0105959)
Supplement: Figure S2 — Accumulation of Solexa total tags in ‘Jingxiu’ and ‘Jingyan’ grape skins. (DOC) [file pone.0105959.s002.doc]

**Figure S2**. Accumulation of Solexa total tags in ‘Jingxiu’ and ‘Jingyan’ grape skins under conditions of sunlight exposure and sunlight exclusion.
