# Supplementary material for: Genome-Wide Transcriptional Profiles of the Berry Skin of Two Red Grape Cultivars (Vitis vinifera) in Which Anthocyanin Synthesis Is Sunlight-Dependent or -Independent
Source: PLoS One. 2014 Aug 26;9(8):e105959. doi: 10.1371/journal.pone.0105959 (PMC4144973; doi:10.1371/journal.pone.0105959)
Supplement: Table S1 — Numbers of down-regulated or up-regulated genes with log2 ratio ≥2 in grape berry skin. (DOC) [file pone.0105959.s003.doc]

**Table S1.** Numbers of down-regulated (down) or up-regulated (up) genes with log2 ratio ≥ 2 at *P* < 0.0001 in sunlight-excluded and sunlight-exposed berry skins of ‘Jingxiu’ (X) and ‘Jingyan’ (Y) grapes, based on MapMan classifications.

| Bin | Bin name | Xup | Xup-  Yup | Xup-  Ydown | Xdown | Xdown-  Yup | Xdown-  Ydown | Yup | Ydown | Total | Percentage  (%)* |
| --- | --- | --- | --- | --- | --- | --- | --- | --- | --- | --- | --- |
| 1 | photosynthesis | 21 | 0 | 9 | 2 | 0 | 7 | 3 | 9 | 51 | 1.5 |
| 2 | major CHO metabolism | 16 | 0 | 0 | 0 | 0 | 1 | 2 | 0 | 19 | 0.6 |
| 3 | minor CHO metabolism | 13 | 3 | 3 | 0 | 0 | 0 | 1 | 2 | 22 | 0.7 |
| 4 | glycolysis | 7 | 1 | 0 | 0 | 0 | 0 | 2 | 1 | 11 | 0.3 |
| 5 | fermentation | 5 | 1 | 0 | 1 | 0 | 0 | 0 | 0 | 7 | 0.2 |
| 6 | gluconeogenesis/glyoxylate cycle | 1 | 0 | 0 | 0 | 0 | 0 | 0 | 0 | 1 | 0.0 |
| 7 | oxidative pentose phosphate pathway | 6 | 1 | 0 | 0 | 0 | 0 | 0 | 0 | 7 | 0.2 |
| 8 | TCA cycle/org. acid transformations | 8 | 2 | 1 | 1 | 0 | 0 | 1 | 0 | 13 | 0.4 |
| 9 | mitochondrial electron transport/ATP synthesis | 17 | 0 | 0 | 0 | 1 | 0 | 1 | 1 | 20 | 0.6 |
| 10 | cell wall | 32 | 1 | 6 | 7 | 2 | 1 | 5 | 6 | 60 | 1.8 |
| 11 | lipid metabolism | 59 | 0 | 6 | 2 | 0 | 1 | 3 | 7 | 78 | 2.3 |
| 12 | nitrogen metabolism | 6 | 0 | 0 | 0 | 0 | 0 | 1 | 0 | 7 | 0.2 |
| 13 | amino acid metabolism | 43 | 1 | 5 | 0 | 2 | 1 | 4 | 3 | 59 | 1.7 |
| 14 | sulphur assimilation | 1 | 0 | 0 | 0 | 1 | 0 | 0 | 0 | 2 | 0.1 |
| 15 | metal handling | 9 | 4 | 4 | 0 | 0 | 0 | 3 | 1 | 21 | 0.6 |
| 16 | secondary metabolism | 41 | 2 | 5 | 7 | 7 | 2 | 15 | 8 | 87 | 2.6 |
| 17 | hormone metabolism | 75 | 4 | 3 | 3 | 3 | 5 | 5 | 3 | 101 | 3.0 |
| 18 | cofactor and vitamin synthesis | 11 | 0 | 0 | 0 | 0 | 1 | 0 | 1 | 13 | 0.4 |
| 19 | tetrapyrrole synthesis | 3 | 1 | 0 | 0 | 0 | 0 | 1 | 2 | 7 | 0.2 |
| 20 | stress | 69 | 2 | 6 | 7 | 5 | 2 | 13 | 8 | 112 | 3.3 |
| 21 | redox | 35 | 3 | 3 | 1 | 0 | 0 | 0 | 3 | 45 | 1.3 |
| 22 | polyamine metabolism | 4 | 0 | 1 | 0 | 0 | 0 | 0 | 0 | 5 | 0.1 |
| 23 | nucleotide metabolism | 19 | 2 | 0 | 0 | 0 | 0 | 0 | 0 | 21 | 0.6 |
| 24 | biodegradation of xenobiotics | 3 | 0 | 0 | 0 | 1 | 0 | 3 | 0 | 7 | 0.2 |
| 25 | C1 metabolism | 4 | 1 | 0 | 0 | 0 | 0 | 0 | 0 | 5 | 0.1 |
| 26 | miscellaneous enzyme families | 130 | 12 | 9 | 8 | 8 | 7 | 38 | 16 | 228 | 6.8 |
| 27 | RNA | 322 | 13 | 13 | 4 | 4 | 5 | 44 | 13 | 418 | 12.4 |
| 28 | DNA | 31 | 0 | 1 | 1 | 0 | 1 | 7 | 3 | 44 | 1.3 |
| 29 | protein | 526 | 27 | 20 | 7 | 8 | 4 | 51 | 24 | 667 | 19.8 |
| 30 | signalling | 113 | 2 | 8 | 5 | 4 | 2 | 24 | 4 | 162 | 4.8 |
| 31 | cell | 104 | 3 | 2 | 1 | 0 | 2 | 8 | 7 | 127 | 3.8 |
| 33 | development | 43 | 4 | 6 | 0 | 2 | 0 | 8 | 3 | 66 | 2.0 |
| 34 | transport | 101 | 11 | 6 | 8 | 4 | 5 | 29 | 7 | 171 | 5.1 |
| 35 | not assigned | 505 | 22 | 32 | 17 | 9 | 15 | 72 | 40 | 712 | 21.1 |
| Total |  | 2383 | 123 | 149 | 82 | 61 | 62 | 344 | 172 | 3376 |  |

Xup-Yup and Xdown-Ydown indicate that genes were, respectively, up-regulated and down-regulated in both ‘Jingxiu’ and ‘Jingyan’.

Xup, Xdown, Yup, Ydown indicate that genes were up-regulated and down-regulated in either ‘Jingxiu’ or ‘Jingyan’.

Xup-Ydown and Xdown-Yup indicate that genes were regulated contrarily in ‘Jingxiu’ and ‘Jingyan’.

* Percentage of each MapMan Bin of the total 3376 genes.
