# Supplementary material for: Genome-Wide Transcriptional Profiles of the Berry Skin of Two Red Grape Cultivars (Vitis vinifera) in Which Anthocyanin Synthesis Is Sunlight-Dependent or -Independent
Source: PLoS One. 2014 Aug 26;9(8):e105959. doi: 10.1371/journal.pone.0105959 (PMC4144973; doi:10.1371/journal.pone.0105959)
Supplement: Table S3 — Forward (F) and reverse (R) primers and expected amplicon sizes of genes for qRT-PCR. (DOC) [file pone.0105959.s005.doc]

**Table S3.** Forward (F) and reverse (R) primers and expected amplicon sizes of genes for qRT-PCR.

| Gene | Primers | Amplicon size (bp) |
| --- | --- | --- |
| GSVIVT01032968001 | F 5’-CTCCAAATAATGGTTGTC-3’  R 5’-CAACTAACGTTTCTCAGAA-3’ | 132 |
| GSVIVT01024419001 | F 5’-GGGATGGTAATGGCTGTGG-3’  R 5’-ACATGGGTGGAGAGTGAGTT-3’ | 151 |
| GSVIVT01035256001 | F 5’-CGAGGGCGATTGTGAGGTA-3’  R 5’-TTCCACTTCCAGCCATTGAT-3’ | 110 |
| GSVIVT01027811001 | F 5’-ACCGGACGTTACAACCATATC-3’  R 5’- TCCGTAACTGGGTTTTTCTCA-3’ | 111 |
| GSVIVT01027182001 | F 5’-AGATCAACTGGTTATGCTTGCT-3’  R 5’-AACACAAATGTACATCGCACAC-3’ | 190 |
| GSVIVT01022659001 | F 5’-TAGTCACCACTTCAAAAAGG-3’  R 5’-GAATGTGTTTGGGGTTTATC-3’ | 65 |
| GSVIVT01016705001 | F5'-TACTCTCTCTCTCTCCGCCT-3’  R5'-TACCCACCTTTCGCAATC-3’ | 151 |
| GSVIVT01009934001 | F5'-GAAGACAACGAAGGGAATG-3’  R5'-GTTATGAATACGACGGCG-3’ | 90 |
| GSVIVT01030511001 | F5'-GGCAGTGATGATTGTAAGG-3’  R5'-TATGAAGTGGATGGCTGG-3’ | 187 |
| GSVIVT01017738001 | F5'-ATTCTACGCTCCTTTATTAGGGTTC-3’  R5'-TTCCGCTCATTCATCGTC-3’ | 102 |
| GSVIVT01021502001 | F5'-CAGACCTGGTGGTTTTCCTATCA-3’  R5'-AGAGCGAACGCACATTTGG-3’ | 135 |
| GSVIVT01015070001 | F5'-ATAACAGCGAATGGGAGT-3’  R5'-CATCACTGAAGCAGAAGTT-3’ | 94 |
| GSVIVT01003780001 | F5’-CACATCAAATGACATCGCAC-3’  R5’-ACACACCGTGGCTGAATCT-3’ | 180 |
| GSVIVT01018273001 | F5'-AACAAAGGCAAGCAGAGC-3’  R5'-TGGGAACAGCGAGAATCT-3’ | 121 |
| GSVIVT01009033001 | F5'-CCCACAGGGAGAATTTTTCA-3’  R5'-TCCCCAGCTTCTAATGGGTA-3’ | 195 |
| GSVIVT01028882001 | F 5’-GCAAACAACAGAGAGGATGC-3’  R 5’-AGACCTCGACAATGATCTTAC-3’ | 136 |
